# *Borrelia recurrentis* Employs a Novel Multifunctional Surface Protein with Anti-Complement, Anti-Opsonic and Invasive Potential to Escape Innate Immunity

**DOI:** 10.1371/journal.pone.0004858

**Published:** 2009-03-24

**Authors:** Sonja Grosskinsky, Melanie Schott, Christiane Brenner, Sally J. Cutler, Peter Kraiczy, Peter F. Zipfel, Markus M. Simon, Reinhard Wallich

**Affiliations:** 1 Infectious Immunology, Institute for Immunology, University of Heidelberg, Heidelberg, Germany; 2 School of Health and Bioscience, University of East London, Stratford, London, United Kingdom; 3 Institute of Medical Microbiology and Infection Control, University Hospital of Frankfurt, Frankfurt/Main, Germany; 4 Department of Infection Biology, Leibniz-Institute for Natural Products Research, Jena, Germany; 5 Metschnikoff Laboratory, Max-Planck-Institute for Immunobiology, Freiburg, Germany; University of California Merced, United States of America

## Abstract

*Borrelia recurrentis*, the etiologic agent of louse-borne relapsing fever in humans, has evolved strategies, including antigenic variation, to evade immune defence, thereby causing severe diseases with high mortality rates. Here we identify for the first time a multifunctional surface lipoprotein of *B. recurrentis*, termed HcpA, and demonstrate that it binds human complement regulators, Factor H, CFHR-1, and simultaneously, the host protease plasminogen. Cell surface bound factor H was found to retain its activity and to confer resistance to complement attack. Moreover, ectopic expression of HcpA in a *B. burgdorferi* B313 strain, deficient in Factor H binding proteins, protected the transformed spirochetes from complement-mediated killing. Furthermore, HcpA-bound plasminogen/plasmin endows *B. recurrentis* with the potential to resist opsonization and to degrade extracellular matrix components. Together, the present study underscores the high virulence potential of *B. recurrentis*. The elucidation of the molecular basis underlying the versatile strategies of *B. recurrentis* to escape innate immunity and to persist in human tissues, including the brain, may help to understand the pathological processes underlying louse-borne relapsing fever.

## Introduction

*B. recurrentis*, the causative agent of louse-borne relapsing fever (LBRF) is transmitted to humans, its reservoir host, by contamination of abraded skin with either hemolymph or feces of an infected human body louse (*Pediculus humanus humanus*) [1], [2]. The last century has seen multiple epidemics of LBRF in Europe, with high mortality rates up to 40%. However, currently epidemic outbreaks of LBRF are only reported from Africa [3], [4]. LBRF is a systemic inflammatory disease, characterized by one to five relapses of fever and a massive spirochetemia [5]. If treated with antibiotics, the mortality is reduced to 2–6%, however, patients often suffer from Jarish-Herxheimer reactions [6].

In order to survive in human tissues, including the blood, *B. recurrentis* has to escape innate and adaptive immune defence processes. Complement acts as an important part of host innate immunity, which is essential for recognition and elimination of microbes. However, pathogenic organisms can evade complement attack that is either mediated by the acquisition of host regulators to the surface of the pathogen or by expression of endogenous complement regulators [7]. An increasing number of pathogenic microbes utilize host complement regulators for immune evasion and for down-regulation of complement activation. In fact we and others have recently demonstrated that tick-borne pathogens, *B. hermsii* and *B. burgdorferi*, are able to bind complement regulatory proteins, i.e. factor H (CFH), CFH-like protein 1 (CFHL-1) and CFH-related protein 1 (CFHR-1), via their surface lipoproteins CRASP-1, thereby conferring resistance to complement attack [8], [9], [10], [11], [12]. Surface bound CFH controls complement activation by accelerating the decay of the C3 convertase of the alternative pathway and by inactivating newly formed C3b [13], [14]. CFH represents the main human fluid phase regulator of the alternative pathway of complement activation and belongs to the factor H protein family, consisting of seven structurally related proteins in humans, including Factor H-related proteins (CFHRs) [15]. All factor H protein family members are composed of short consensus repeats (SCRs) [16]. Among those, CFHR-1 was recently shown to inhibit complement activation by interfering with C5 convertase and terminal complex formation (unpublished data).

Another common strategy of pathogens to circumvent the immune system and to persist is based on their capability to exploit host plasma derived proteases, in particular human plasminogen/plasmin (PLG) in order to breach tissue barriers. Recently, we have identified and characterized a number of PLG-binding outer surface lipoproteins in related spirochetes, including *B. burgdorferi* and *B. hermsii*. *B. burgdorferi* was shown to bind PLG via a multitude of lipoproteins, such as CRASP-3 (ErpP), CRASP-4 (ErpC), CRASP-5 (ErpA), OspA and as yet to be defined molecules [17], [18]. *B. hermsii*, on the other hand, was found to interact with PLG via a 21-kDa lipoprotein, which also binds CFH via distinct, non-overlapping domains [9]. Surface bound PLG is subsequently processed via urokinase-type plasminogen activator (uPA) to active plasmin, a broad-spectrum serine protease, capable of degrading constituents of the extracellular matrix and basement membranes [19], [20]. The fact that surface-bound PLG retains its proteolytic activity, even in the presence of serum inhibitors, suggests this process as a suitable means of pathogens to readily disseminate and to persist in the host.

CFH- and C4b-binding proteins of *B. recurrentis* have been proposed [21], but so far these proteins have not been isolated. Here we provide evidence that *B. recurrentis* express a novel multifunctional surface lipoprotein, which by exploiting host proteins confers resistance to both, complement attack and opsonization and simultaneously acquires an increased potential to invade host tissues.

## Materials and Methods

### Bacterial strains and growth conditions

Relapsing fever spirochetes *B. recurrentis* strain A1 and A17, *B. hermsii* (ATCC35209) strain HS1 and the Lyme disease spirochete *B. burgdorferi* strain B313, which is a clonal mutant of B31 lacking all linear and circular plasmids with the exception of cp32-1, cp32-2, cp32-4, cp26 and lp17, were cultivated in BSK-H complete medium (PAN Biotech, Aidenbach, Germany) supplemented with 5% rabbit serum (Cell Concept, Freiburg, Germany) at 30°C [22]. Bacteria were harvested by centrifugation and washed with phosphate-buffered saline. The density of spirochetes was determined using dark-field microscopy and a Kova counting chamber (Hycor Biomedical, Garden Grove, CA). *E. coli* JM109 were grown at 37°C in LB medium.

### Isolation and cloning of HcpA, construction of expression plasmids and production of recombinant proteins

Isolation of the CFH binding protein of *B. recurrentis* was carried out by co-immunoprecipitation. Protein G sepharose beads (Amersham Bioscience, Freiburg, Germany) were loaded with polyclonal anti-factor H antibody (Calbiochem, Schwalbach, Germany) and purified human factor H (Calbiochem). Subsequently, beads were incubated overnight at 4°C with whole cell lysates of *B. recurrentis* A1. Immunoprecipitates were separated by SDS-PAGE and visualized by staining with colloidal Coomassie (Pierce/Thermofisher, Bonn, Germany). The selected protein band of approximately 17 kDa was cored from the gel and subjected to mass spectrometric analysis. The identified peptides matched an open reading frame of 525 bp of the *B. recurrentis* A1 genome, designated *hcpA*.

The gene encoding HcpA was amplified by PCR using primers BrF and BrR (Table 1), cloned into pGEM-T Easy vector (Promega, Mannheim, Germany) and sequenced by using the BigDye terminator cycle sequencing kit (PE Applied Biosystems). The resulting plasmid pGEM-Br was used as template for construction of expression plasmids by PCR amplification. For recombinant full-length HcpA protein, primers BrBam and BrSal were used, for N- and C-terminal deletion mutants, these primers were applied in combination with Δ48Bam, Δ78Bam, Δ145Sal or Δ115Sal (Table 1) resulting in recombinant proteins HcpA Δ48-175, Δ78-175, Δ18-145 and Δ18-115, respectively. After digestion with restriction enzymes BamHI and SalI, PCR fragments were ligated in frame into the His_6_-tag encoding sequence into vector pQE-30Xa (Qiagen, Hilden, Germany). For expression of N-terminal His-tagged fusion proteins the plasmids were transformed into *E.coli* strain JM109 and recombinant proteins were purified as recommended by the manufacturer (Qiagen).

**Table 1:** Oligonucleotides used in this study.

Primer	Sequence (5′ to 3′)	purpose of use
BrF	TTC AGA AGT GGA GCA ATC	amplification of *hcpA* gene
BrR	ACA TAA TAA ACG AAT TTT AAT CTA TG	amplification of *hcpA* gene
BrBam	CAT TAT TGG ATC CGA ACT CTT AAG C	generation of expression plasmid
BrF1	AAA CAG GCT CAA TCA GCT C	amplification of *hcpA* gene
BrR1	TCT AGA TCA TTA AAG TTT GCA G	amplification of *hcpA* gene
BrSal	ACA TAA TAA ACG TCG ACT AAT CTA TG	generation of expression plasmid
Δ48Bam	CAG GCT GGA TCC TTT TTT CAA GAA TC	construction of deletion mutants
Δ78Bam	AA GAT GGA TCC CAA AAG ACA TTA AAC	construction of deletion mutants
Δ145Sal	TA TAA GTC GAC TTA TAT AGA GTT TAT TG C	construction of deletion mutants
Δ115Sal	AA AGA GTC GAC TTA GAT AAA CTT TAT CG	construction of deletion mutants
BrTBam	TTC AGA AGT GGA TCC ATC	construction of pBR
BrTSph	GGT AAT TAA TTA TGC ATG CAA TAA TTA ATT ATT TCA AG	construction of pBR

### Expression of recombinant proteins of CFH, CFHL-1 and CFHR-1

Deletion constructs of CFH (CFH_SCR1-7_, CFH_SCR8-20_, CFH_SCR15-20_, CFH_SCR15-19_) and CFHR-1 were expressed in *Spodoptera frugiperda* Sf9 insect cells infected with a recombinant baculovirus. The cloning, expression and purification have been described previously [14], [23]. The CFH deletion mutant CFH_SCR19-20_ was amplified by PCR, ligated in frame into pQE-30Xa vector and expressed as fusion protein with an N-terminal His_6_-tag. Expression and purification was done as recommended by the manufacturer (Qiagen).

### SDS-PAGE, Ligand affinity blot and ELISA

Borrelial whole cell lysates (15 µg) or purified recombinant HcpA proteins (200 ng) were subjected to Tris/Tricine-SDS-PAGE under reducing conditions and transferred to nitrocellulose as previously described [24]. For ELISA using non-denatured recombinant proteins, microtiter plates (MaxiSorp, Nunc) were coated with HcpA or the deletion mutants (100 µl; 1 µg/ml) for 2 h at room temperature. The wells were washed with PBS/0.05%Tween, blocked with PBS/5% BSA and incubated with CFH (10 µg/ml), 50% normal human serum (NHS) or PLG (20 µg/ml) for 1 h at RT. After washing, HcpA bound proteins were detected by goat anti-CFH (Calbiochem) or goat anti-PLG (Acris) antibodies or CFHR-1 specific mouse mAb JHD8 followed by peroxidase-conjugated rabbit anti-goat IgG (Dianova) or sheep anti-mouse secondary antibody (GE Healthcare), respectively. Substrate reaction was performed with o-phenyldiamine dihydrochloride (Sigma-Aldrich) and absorbance was measured at 492 nm. For competition inhibition assay, HcpA (2 µg/ml) was coated on microtiter plates. To analyze the ability of PLG to inhibit the binding of CFH to HcpA, plates were incubated simultaneously with constant amounts of CFH (3 µg/ml) and different amounts of PLG (0.001–100 µg/ml). The ability of CFH to inhibit the binding of PLG to HcpA was determined by adding constant amounts of PLG (10 µg/ml) with different amounts of CFH (0.001–100 µg/ml). HcpA bound CFH and PLG were detected as described above. For detection of purified recombinant HcpA full-length protein and deletion mutants, the anti His_6_-tag monoclonal mouse antibody (Calbiochem) was used.

### Immunofluorescence analysis

Spirochetes (1×10^7^) were washed with Tris buffer (30 mM Tris, 60 mM NaCl, pH 7.4) and incubated with mAb directed against HcpA (BR-1) for 1 h at RT. For detection of CFH-binding cells were treated with purified human CFH for 1 h at RT followed by incubation with CFH-specific mAb JHD7. Spirochetes were then washed with Tris buffer/0.1% BSA, spotted on coverslips and allowed to air-dry for 1 h. After methanol fixation, samples were dried for 15 min and incubated for 1 h in a humidified chamber with Cy3-labeled rabbit anti-mouse IgG (1/200, Dianova). Cells were visualized at a magnification of 1000× using a Nikon Eclipse 90i upright automated microscope and images were obtained using a Nikon DS-1 QM sensitive black and white CCD camera at a resolution of 0.133 µm/pixel.

### Flow cytometry

Briefly, 10^7^ *B. recurrentis* A1 and A17, *B. hermsii* HS1 and *B. burgdorferi* B313 cells were washed twice with PBS, blocked for 15 min at RT with PBS/10%BSA, and incubated with 10 µg/ml of biotinylated CFH in FACS-buffer (PBS/1%BSA) for 1 h at RT. As a negative control, spirochetes were incubated with same concentration of biotinylated BSA. Cells were washed intensively for three times and stained with phycoerythrin (PE) labeled streptavidin (Bio-Rad). Cells were then fixed with 1% paraformaldehyde overnight and analyzed on a FACS-Calibur (BD Biosciences) and CellQuest software (BD).

### In situ protease treatment of spirochetes

Cells of *B. recurrentis* strain A1 were treated with proteases using a modified, previously described method [25]. Briefly, intact borrelial cells were incubated with either proteinase K or trypsin and whole-cell protein preparations were separated by SDS-PAGE (13%) as described [26].

### Surface plasmon resonance analysis

Protein-protein interactions were analyzed using surface plasmon resonance with a Biacore 3000 instrument (Biacore AB), as described elsewhere [27].

### Cofactor asssay

Cofactor activity of CFH bound to immobilized HcpA or intact *B. recurrentis* A1 cells was analyzed by measuring factor I-mediated conversion of C3b to inactive C3b (iC3b) [28].

### Substrate assay for plasmin bound to spirochetes and HcpA

Intact *B. recurrentis* A1 cells (1×10^8^) were incubated with 10 µg PLG (Chromogenix) in the presence or absence of 100 mM tranexamic acid for 30 min at 30°C in Eppendorf tubes. Following two washes, *B. recurrentis* were resuspended in 50 µl assay buffer (30 mM Tris, 60 mM NaCl, pH 7.4), transferred to microtiter plates and uPA (25 ng, Chemicon Int., Hampshire, UK) as well as plasmin substrate D-Val-Leu-Lys 4-nitroanilide dihydrochloride (20 µg, S-2251, Sigma-Aldrich, Taufkirchen, Germany) was added. The absorbance change at 405 nm was monitored for up to 6 hours directly in the plates. Similarly, HcpA (0.2 µg) was coated on microtiter plates and after blocking, PLG in the presence or absence of 100 mM tranexamic acid was added, incubated for 10 min at 37°C and experiment was carried out as described above. For plasmin-dependent degradation of fibrinogen, HcpA (0.2 µg) coated microtiter plates were incubated with PLG. Subsequently, fibrinogen (500 ng, Calbiochem) and uPA (10 ng) were added and incubated at 37°C for 0.5, 1, 2, 4 and 6 h. Reaction mixtures were then separated by SDS-PAGE, transferred to nitrocellulose and probed with rabbit anti-fibrinogen Ab (Calbiochem) and peroxidase-conjugated goat anti-rabbit secondary antibody (Dianova) for detection of fibrinogen degradation products.

### C3b deposition and degradation on borrelial surface

Anti-opsonic properties of plasmin bound to the borrelial surface were investigated by a C3b deposition and degradation assay based on whole-cell ELISA. Intact *B.recurrentis*, *B. burgdorferi* B313 and transformed *B. burgdorferi* B313/pBR spirochetes (10^7^/well) were washed, resuspended in PBS and immobilized onto microtiter plates (MaxiSorp, Nunc) overnight at 4°C. After washing with PBS/0.05%Tween, wells were blocked with PBS/0.1% gelatine for 1 h at RT and incubated with 10% normal human serum (NHS) for 30 min at 37°C. *B. burgdorferi* B313 and B313/pBR were processed instantly, whereas *B. recurrentis* cells were incubated with PLG (10 µg) in the presence or absence of the lysine analogue tranexamic acid (TA); as a negative control, cells were treated with buffer. Bound PLG was activated by uPA for 3 h at 37°C. Deposited C3b was then detected by incubation with biotinylated rabbit anti-human C3c IgG (Dako) followed by peroxidase-conjugated streptavidin (Amersham Bioscience) and analyzed as described above.

### Construction of a shuttle vector for transformation of B. burgdorferi B313

The HcpA encoding *hcpA* gene including its native promoter region was amplified by PCR using primers BrTBam and BrTSph. The resulting amplicon was cloned into pBSV2 yielding shuttle vector pBR [29]. Transformation of *B. burgdorferi* B313 and characterization of transformants was previously described [30]. Expression of HcpA of post-transformation *B. burgdorferi* B313 was determined by Western blot using mAb BR-1 and sheep anti mouse peroxidase-conjugated secondary Ab.

High-passage, non-infectious *B. burgdorferi* strain B313 were grown in 100 ml BSK medium and harvested at mid exponential phase (10^8^ cells/ml). Electrocompetent cells were prepared as described previously [30] with slight modifications. Briefly, 50 µl aliquots of competent *B. burgdorferi* strain B313 cells were electroporated at 12.5 kV/cm in 2-mm cuvettes with 10 µg of plasmid DNA. For control purpose *B. burgdorferi* strain B313 cells also were transformed with pBSV2 vector alone. Cells were immediately diluted into 10 ml BSK medium and incubated without antibiotic selection at 30°C for 48 to 72 h. Bacteria were then diluted into 100 ml BSK medium containing kanamycin (30 µg/ml) and 200 µl aliquots were plated into 96-well cell culture plates (Corning) for selection of transformants. After three weeks, wells were evaluated for positive growth by color change of the medium, confirmed by dark-field microscopy for the presence of motile spirochetes.

The *hcpA* genes of transformed *B. burgdorferi* B313 strain was detected by PCR using primers BrF1 and BrR1 (Table 1) and expression of the HcpA was determined by Western blot and ligand affinity blot analysis.

### Serum susceptibility testing of Borrelia strains

The serum susceptibility of *B. recurrentis* A1, *B. burdorferi* B313 and transformed *B. burgdorferi* B313 was assessed using a survival assay. Cells grown to mid-logarithmic phase were harvested, washed and 3×10^7^ spirochetes were resuspended in BSK-H medium (PAN) supplemented with either 50% or 25% NHS or heat inactivated serum (hiNHS) as indicated. Cells were incubated in Eppendorf tubes at 30°C for 3 days. At day 0, 1, 2 and 3, cells were washed in 0.85% NaCl, transferred to microtiter plates and incubated with SYTO9 (Molecular Probes, Invitrogen) as recommended by the manufacturer. Subsequently, relative growth of spirochetes was determined measuring the fluorescence intensity at 485 nm/530 nm on a microtiter plate reader (Victor^2^ plate reader, Perkin Elmer).

### Production of monoclonal antibodies

Monoclonal antibodies directed against HcpA were generated by immunization of Balb/c mice with the respective purified recombinant protein according to a method as described elsewhere [31]. For specific detection of CFHR-1, mAb JHD8 and for specific detection of His-tagged CFH deletion mutant SCR19-20, mAb JHD7 were generated accordingly.

### Nucleotide sequence deposition

The *hcpA* gene sequence encoding HcpA has been deposited in the EMBL/GenBank databases under the following accession numbers: FM946025.

### Statistical analysis

Statistics were analyzed with the unpaired Student's *t*-test, P values less than 0.01 were considered significant.

## Results

### B. recurrentis spirochetes acquire complement regulators CFH and CFHR-1

**Figure 1:**
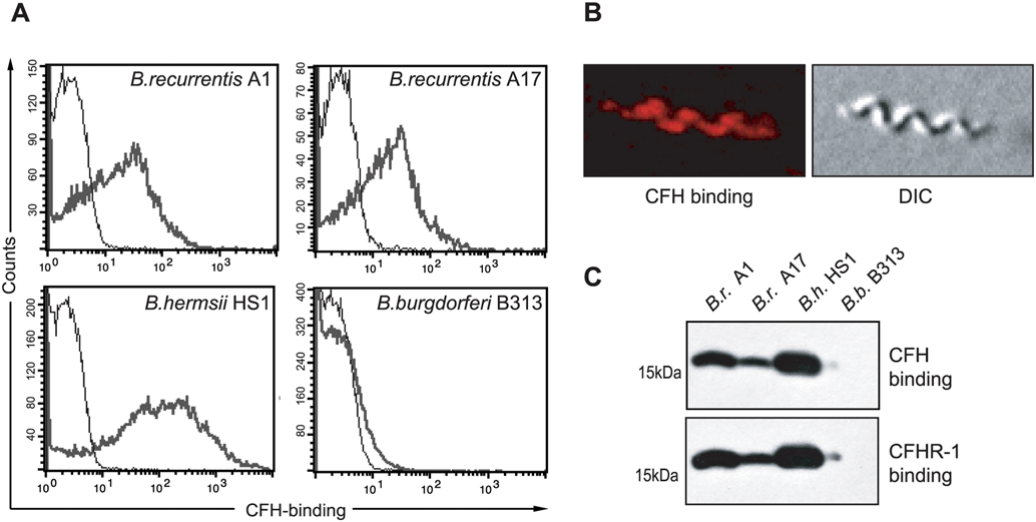
Binding of CFH and CFHR-1 to the spirochetal surface. Binding of CFH to intact *B. recurrentis* cells was analyzed by flow cytometry and immunofluorescence microscopy. (A) Spirochetes were incubated with biotinylated purified human CFH (bold lines) or as a negative control with biotinylated BSA followed by PE-labeled streptavidin. *B.hermsii* HS1 and *B. burgdorferi* B313 were included as controls. (B) Cells were incubated with purified human CFH followed by the CFH-specific mAb JHD7 and a Cy3-conjugated anti-mouse IgG. Images were obtained employing epifluorescence microscopy. On the right panel the corresponding differential interference contrast image (DIC) is depicted. (C) Whole cell lysates of *B. recurrentis* strain A1 and A17 (*B.r.* A1 and *B.r.* A17) were separated by Tris/Tricine SDS-PAGE, transferred to nitrocellulose membrane and incubated with normal human serum. CFH binding was detected employing CFH specific mAb JHD7 and binding of CFHR-1 was analyzed using specific mAb JHD8. For control, cell lysates of *B. hermsii* HS1 (*B.h* HS1) and *B. burgdorferi* B313 (*B.b* B313) were included.

In light of previous experience that *B. hermsii* and *B. burgdorferi* can specifically bind CFH via their outer surface lipoproteins [8], [11], [32], *B. recurrentis* spirochetes were incubated with biotinylated human CFH and analysed by flow cytometry. As seen in Figure 1A, both, *B. recurrentis* strains (A1 and A17) and *B. hermsii* HS1, but not *B. burgdorferi* B313, which lacks the CFH-binding lipoprotein, were able to bind CFH. In addition, biotinylated control protein, BSA, did not bind to any of the borrelial strains (Fig. 1A). Binding of CFH to intact and viable *B. recurrentis* spirochetes was confirmed by microscopy, using a CFH-specific mAb, JHD7 (Fig. 1B). By applying ligand affinity blot analysis for detection of CFH- and CFHR-1 binding molecules, a protein of approximately 17 kDa was detected in *B. recurrentis* but not in *B. burgdorferi* B313 (Fig. 1C), termed human complement regulator(s) and plasminogen binding protein A (HcpA) [8], [9].

### HcpA is a CFH, CFHR-1 and PLG binding protein

**Figure 2:**
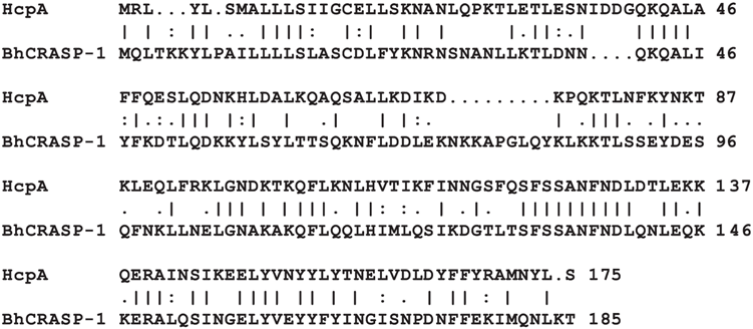
HcpA exhibits 54% amino acid sequence similarity to BhCRASP of *B. hermsii* HS1.

**Figure 3:**
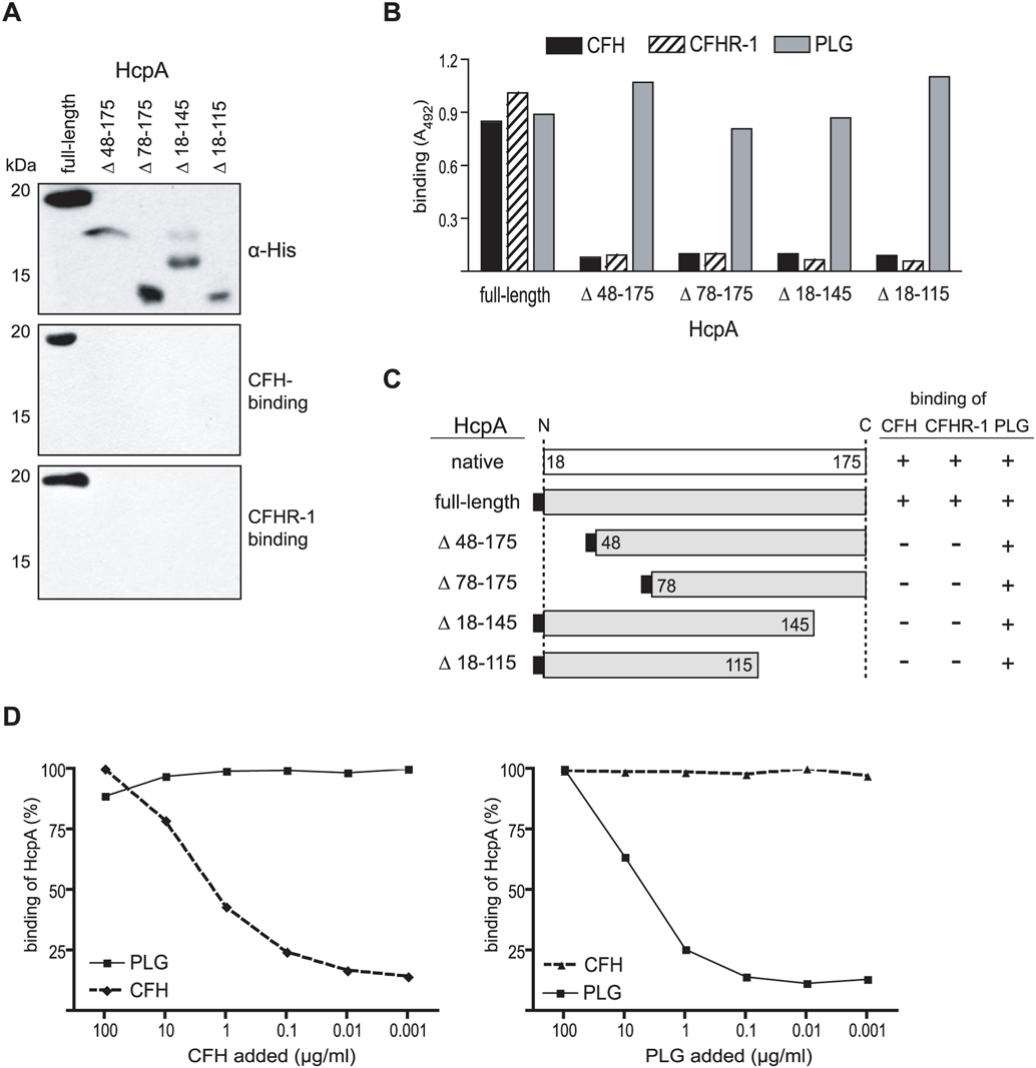
Binding of CFH, CFHR-1 and PLG to HcpA. (A) Purified recombinant HcpA protein and various deletion mutants were separated by SDS-PAGE and transferred to nitrocellulose. Membranes were incubated with a monoclonal anti-His-tag mAb (upper panel). CFH and CFHR-1 binding capabilities were analyzed by ligand affinity blotting utilizing normal human serum and goat anti-CFH immune serum (middle panel) or the CFHR-1 specific mAb JHD8 (lower panel). (B) Microtiter plates were coated with full-length recombinant HcpA and the indicated deletion mutants, respectively, and incubated with CFH, normal human serum (as source for CFHR-1) or PLG. Binding was detected using goat anti-CFH, the CFHR-1 specific mAb JHD8 or goat anti-PLG immune serum followed by the respective peroxidase-conjugated IgGs. (C) Diagrammatic representation of native and expressed recombinant HcpA proteins and their binding characteristics for serum proteins CFH, CFHR-1 and PLG as determined by ligand affinity blot analysis and ELISA. Numbers refer to amino acid residues. (D) Dose dependent binding of CFH and PLG by HcpA. A competition inhibition assay was performed, adding different amounts of PLG or CFH to inhibit the binding of 2 µg/ml CFH (left panel) or 10 µg/ml PLG (right panel) to immobilized HcpA. Bound CFH and PLG was detected using goat anti-CFH (dashed line) and goat anti-PLG Ab (solid line), respectively, followed by peroxidase-conjugated secondary antibody.

To isolate and further characterize HcpA, whole cell lysates of *B. recurrentis* A1 were incubated with CFH and subsequently treated with a goat anti-CFH immune serum. A resulting co-precipitated protein of 17 kDa was analyzed by mass spectrometry and peptides generated matched an open reading frame of 525 bp on the genome of *B. recurrentis* A1 (unpublished data), designated *hcpA*. Due to the presence of a spirochetal lipobox at its N-terminus HcpA represents a putative outer surface lipoprotein [33]. The deduced amino acid sequence exhibits 54% similarity with the recently identified BhCRASP-1 of *B. hermsii* HS1 (Fig. 2) [8]. A BLAST search detected another protein with significant homology in the genome of *B. turicatae*, indicating that this protein is to be found in other *Borrelia* species. To further elucidate the binding properties of HcpA for complement regulators CFH and CFHR-1, various N- and C-terminal deletion mutants of HcpA were generated. Variants of the encoding *hcpA* gene lacking the hydrophobic leader peptide and the indicated N- or C-terminal regions were cloned and expressed as His-tagged fusion proteins in *E. coli* (Fig. 3). Expression of each protein was confirmed by immunoblot analysis using anti-His antiserum (Fig. 3A).

To assess binding of recombinant HcpA for CFH and CFHR-1, ligand affinity blotting techniques and ELISA were employed in combination with intact HcpA and various deletions thereof. CFH and CFHR-1 only bound to full-length HcpA, but not to any of the deletion mutants. This indicates that long-range intramolecular interactions are involved in the formation of the CFH and CFHR-1 binding site rather than linear peptide sequences (Fig. 3A). Furthermore, full-length and mutant HcpA proteins were analyzed for binding of human PLG using ELISA. Full-length HcpA (residues 18 to 175) as well as the truncated versions bound PLG (Fig. 3B), thus indicating that the binding site for PLG is localized to the central domain of HcpA. Together, these data suggest that CFH and PLG bind to distinct, non-overlapping domains of the HcpA molecule (Fig. 3C). To verify this assumption, competition assays were performed using immobilized HcpA and increasing amounts of PLG or CFH (up to 100 µg/ml) in combination with constant amounts of CFH (2 µg/ml) or PLG (10 µg/ml), respectively. As shown in Fig. 3D, PLG did not compete with CFH for binding to HcpA even at high concentrations (100 µg/ml). Vice versa, using up to 100 µg/ml of CFH no inhibition of PLG binding to HcpA could be observed.

**Figure 4:**
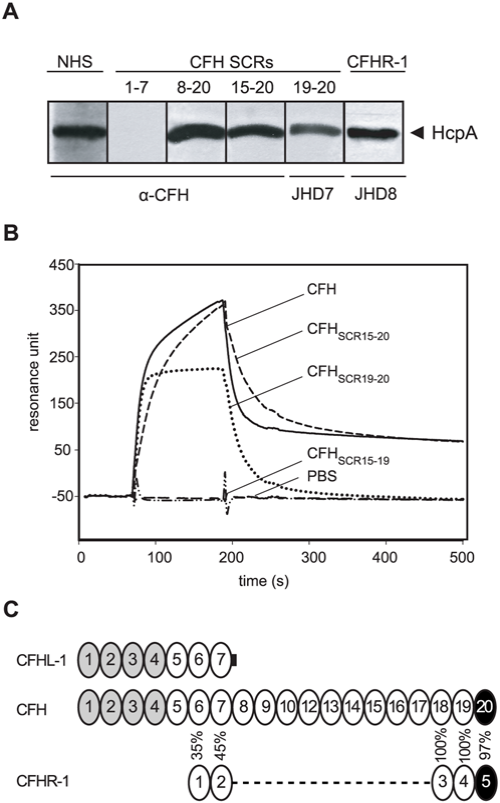
Mapping of the CFH domain interacting with HcpA. (A) Purified recombinant HcpA protein was separated by SDS-PAGE and transferred to nitrocellulose membranes. The membrane strips were incubated with either normal human serum (NHS), recombinant CFHL-1 (CFH_SCR 1-7_), several deletion constructs of CFH (CFH_SCR8-20_, CFH_SCR15-20_, CFH_SCR15-19_, CFH_SCR19-20_) or recombinant CFHR-1 (CFHR-1). Bound proteins were visualized using either polyclonal anti-CFH immune serum (α-CFH) or mAb specific for SCR19-20 (JHD7) or CFHR-1 protein (JHD8), respectively. (B) Binding of CFH and deletion mutants to HcpA as analyzed by surface plasmon resonance. Recombinant HcpA was immobilized to the surface of a sensor chip and CFH or various deletion mutants (CFH_SCR15-20_, CFH_SCR19-20_, CFH_SCR15-19_) were applied in the fluid phase. No binding was detectable for CFH_SCR15-19_ mutant. (C) Schematic representation of the CFH, CFHL-1 and CFHR-1 protein. Complement regulatory domains in SCR1-4 are shown in gray and the HcpA binding region in SCR20 of CFH and the corresponding SCR5 of CFHR-1 are highlighted in black with white fonts. SCR domains are aligned vertically according to their amino acid sequence similarities.

To map the binding domain of CFH that interacts with HcpA, recombinant deletion constructs of CFH representing SCRs 8-10, SCRs 15-10, SCRs 19-20, and SCRs 1-7/FHL-1 were employed. HcpA showed strong binding to CFH and deletion constructs CFH_SCR8-20_, CFH_SCR15-20_, CFH_SCR19-20_, as well as CFHR-1. Construct CFHL-1 (CFH_SCR1-7_) did not bind to HcpA, indicating that the most C-terminal domains (SCR19-20) of CFH are involved in binding (Fig. 4A). We next conducted surface plasmon resonance analyses, a more physiological assay system, to further define the CFH domain interacting with HcpA. CFH and deletion constructs CFH_SCR8-20_, CFH_SCR15-20_ and CFH_SCR19-20_ bound to immobilized HcpA with similar intensity (Fig. 4B). However, in the absence of SCR20, represented by the deletion construct CFH_SCR15-19_, binding to HcpA was completely abrogated (Fig. 4B). As indicated by the schematic representation, domain SCR20 of CFH displays 97% sequence similarity to the SCR5 of CFHR-1 (Fig. 4C) [7]. Therefore it is assumed that the binding region of CFHR-1 for HcpA is located in the C-terminus, accordingly.

### HcpA is exposed on the outer surface of B. recurrentis

**Figure 5:**
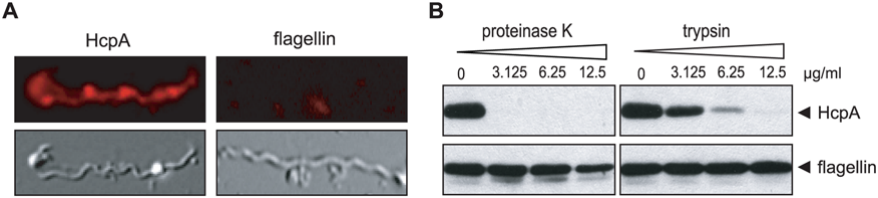
Surface localization of HcpA. (A) Immunofluorescence analysis of *B.recurrentis* A1 after incubation with a mAb specific for HcpA (BR-1) (left panels) or a flagellin-specific mAb (LA21, right panels) followed by rabbit anti-mouse Cy3-conjugated IgG. Corresponding differential interference contrast images are shown in the lower panels. The images were obtained as described above. (B) Proteinase K and trypsin treatment affects surface expression of native HcpA. *B. recurrentis* cells were incubated with the indicated concentrations of proteinase K and trypsin, lysed, immunoblotted, and probed with either anti-HcpA mAb BR-1 (upper panel) or with anti-flagellin mAb LA21 (lower panel).

To determine whether HcpA is exposed on the outer surface of *B. recurrentis* immunofluorescence microscopy was performed. Intact *B. recurrentis* spirochetes were incubated with the HcpA specific mAb BR-1 followed by a Cy3-conjugated secondary antibody (Fig. 5A, left panels). *B. recurrentis* expressed HcpA on its outer surface and the staining showed a patchy distribution. Mouse mAb LA21 directed against the periplasmic flagellin was used as an internal control to confirm that the fragile spirochetal outer membrane was intact (right panels). To further verify surface localization of HcpA, *B. recurrentis* spirochetes were pre-treated with either proteinase K or trypsin, lysed, separated by SDS-PAGE and assayed by Western blotting. Fig. 5B demonstrates a significant reduction of HcpA after 2 h of incubation with trypsin at concentrations ≥12.5 µg/ml, whereas treatment with proteinase K at low concentrations (≥3.125 µg/ml) resulted in complete degradation. Signal intensity observed for flagellin remained unchanged, indicating that periplasmic flagella were not affected by proteolytic digestion. These data strongly suggest that HcpA is exposed on the outer surface of *B. recurrentis*.

### CFH retains cofactor activity when bound to HcpA

**Figure 6:**
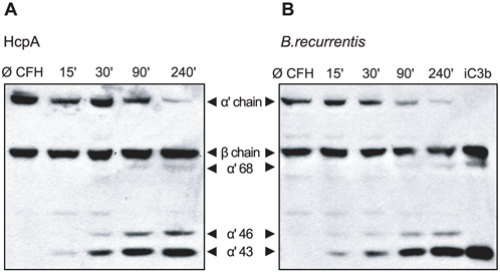
Cofactor activity of CFH bound to either HcpA or intact *B. recurrentis* spirochetes. Functional activity of CFH was analyzed by measuring factor I-mediated conversion of C3b to iC3b. CFH bound to HcpA coated microtiter plates (A) or to the surface of intact *B. recurrentis* spirochetes (B) was incubated with C3b and factor I. Reaction mixtures were separated by SDS-PAGE and transferred to nitrocellulose membrane. C3b degradation products were evaluated by detection of α′-chain cleavage fragments of 68, 46 and 43 kDa using biotinylated rabbit anti-C3c IgG followed by peroxidase-conjugated streptavidin. Purified iC3b was included as control.

To assess whether HcpA-bound CFH maintains its complement regulatory function, cofactor activity of the attached CFH was analyzed by measuring factor I-mediated conversion of C3b to iC3b. To this end, CFH was attached to immobilized HcpA and incubated with C3b and factor I. As shown in Figure 6A, HcpA-bound CFH efficiently mediated C3b conversion as indicated by the appearance of C3b cleavage products (68, 46 and 43 kDa α′-chain). Incubation of immobilized HcpA alone had no effect on C3b conversion under similar conditions. Next we determined whether CFH also retains its cofactor activity after previous binding to *B. recurrentis*. Accordingly, spirochetes were pre-treated with CFH and after intensive washings, incubated with factor I and C3b. Lysates were separated by SDS-PAGE and C3b cleavage products were detected by Western blotting. Surface-bound CFH retained cofactor activity as indicated by the presence of representative C3b inactivation products (Fig. 6B). *B. recurrentis* spirochetes alone did not promote cleavage of C3b demonstrating that *B. recurrentis* lack endogenous C3b cleaving activity and cofactor activity for cleavage. Thus, binding of CFH to the surface of *B. recurrentis* renders them resistant to complement attack.

### Surface bound PLG is processed by host-derived plasminogen activators to plasmin, cleaves fibrinogen and exhibits anti-opsonic activities

**Figure 7:**
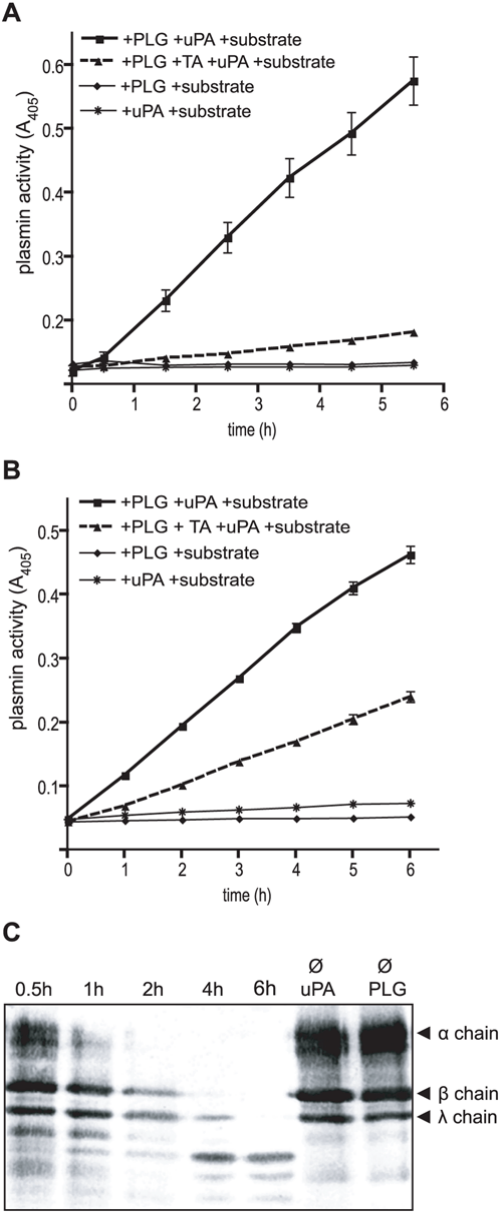
Activation and proteolytic activity of HcpA- and *B. recurrentis*-bound plasmin(ogen). Intact *B. recurrentis* organisms (A) or recombinant HcpA (B) were incubated with PLG. Bound PLG was converted into plasmin by uPA addition and plasmin activity was measured using the chromogenic substrate D-Val-Leu-Lys 4-nitroanilide dihydrochloride (S-2251). uPA mediated PLG activation was inhibited by tranexamic acid (TA). Substrate cleaving was monitored by measurement of the absorbance at 405 nm for up to 6 hrs. Mean of triplicates ± SEM is shown. (C) Degradation of fibrinogen by HcpA-bound plasmin. HcpA coated microtiter plates were incubated with PLG, subsequently fibrinogen and uPA were added. The reaction mixtures were separated by SDS-PAGE, transferred to nitrocellulose and probed with rabbit anti-fibrinogen followed by peroxidase-conjugated IgG for detection of α, β and γ chains (67, 57 and 47 kDa) and the small-size degradation products of fibrinogen.

To assess whether *B. recurrentis*-attached PLG can be processed to active plasmin, spirochetes were pre-treated with PLG and subsequently incubated with the exogenous human plasminogen activator uPA. As shown in Figure 7A, *B. recurrentis*-attached PLG was readily processed by exogenous uPA. In contrast, only marginal plasmin activity was observed in the presence of tranexamic acid, a competitive inhibitor of the lysine-binding site of PLG. These data demonstrate that spirochetal surface-bound PLG is accessible for uPA, and that processed plasmin retains its proteolytic activity. No plasmin was generated from surface-bound PLG in the absence of uPA, indicating that spirochetes do not express endogenous plasminogen activators. Similar findings were observed when recombinant HcpA was coated onto microtiter plates supporting the notion that HcpA-bound PLG can be processed to stable functional active plasmin (Fig. 7B).

The proteolytic activity of HcpA-bound plasmin was further analyzed by its ability to cleave fibrinogen. When incubated with HcpA-bound plasmin, fibrinogen was degraded to low molecular mass fragments, as assessed by Western blotting (Fig. 7C). In the absence of either PLG or uPA, no fibrinogen degradation was observed. Furthermore, the addition of the plaminogen inhibitor tranexamic acid to the reaction mixture abrogated the respective proteolytic activity (data not shown).

**Figure 8:**
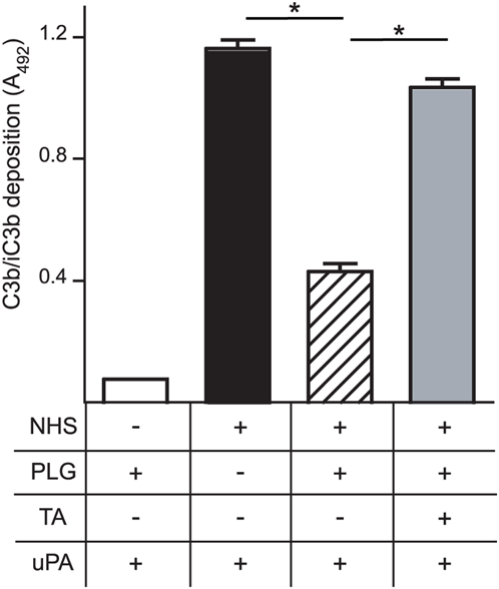
Degradation of deposited C3b by acquiring plasmin on the surface of *B. recurrentis*. Spirochetes were immobilized onto microtiter plates and incubated with 10% NHS as source for C3b. Washed bacteria were treated with PLG in the presence or absence of tranexamic acid (TA) and bound PLG was activated by uPA. Deposited C3b was detected using biotinylated anti-C3c IgG followed by peroxidase-conjugated streptavidin. C3b deposition is expressed as the mean absorbance at 492 nm of triplicates. Error bars indicate±SEM. *, P<0.0001.

Upon activation of complement by bacterial pathogens C3b is covalently attached to the target surfaces and together with the cleavage products, such as iC3b, the C3b molecules opsonize the pathogenic organisms for phagocytosis. To assess whether plasmin bound to HcpA is able to degrade C3b deposited on the cell surface of *B. recurrentis* a C3b deposition and degradation assay based on whole-cell ELISA was performed. Spirochetes were immobilized onto microtiter plates and treated with human serum and PLG. After extensive washing *B. recurrentis*-bound PLG was activated by uPA and deposition of C3b molecules on the spirochetal cell surface was monitored. *B. recurrentis*-bound active plasmin led to a dramatic decrease of C3b molecules on the spirochetal surface when compared to control spirochetes cultured either with buffer alone, or those pre-treated with PLG in the presence of tranexamic acid (Fig. 8). Thus, *B. recurrentis* cell surface-bound plasmin exhibits anti-opsonic properties by cleaving C3b molecules.

### HcpA confers resistance to complement-mediated killing

**Figure 9:**
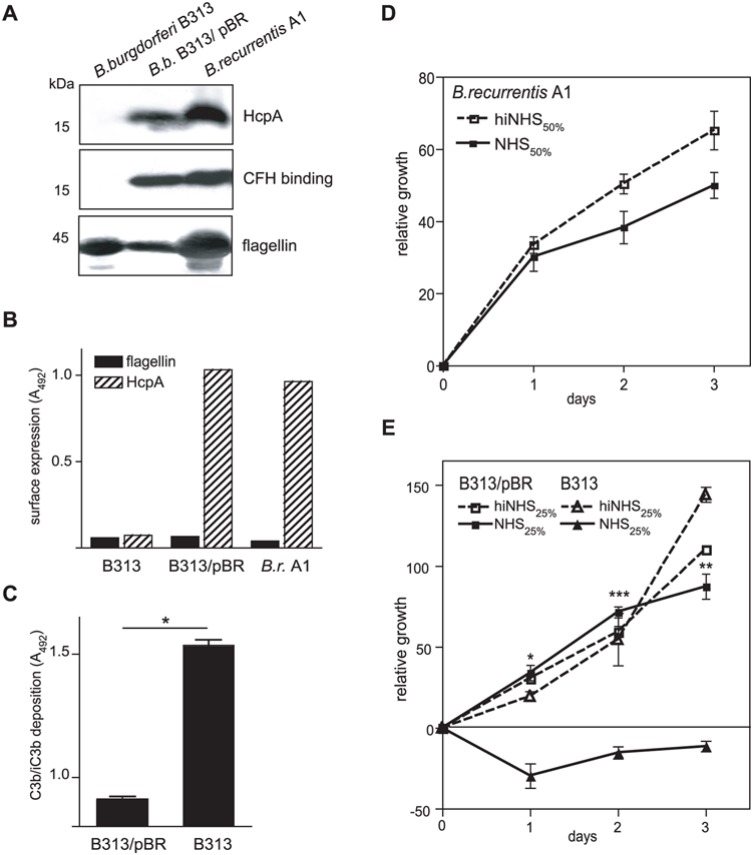
Ectopic expression of HcpA in serum-sensitive *B. burgdorferi* B313. (A) Expression of HcpA by transformed *B. burgdorferi* B313 was assessed using immunoblot analysis. Whole cell lysates were separated by SDS-PAGE, transferred to nitrocellulose and probed with mAb BR-1 (upper panel) or analyzed for CFH binding by incubation with NHS and a CFH-specific mAb (JHD7, middle panel) followed by peroxidase conjugated IgG. For control, a flagellin-specific antibody (LA21) was used (lower panel). (B) Surface expression of HcpA as analyzed by whole cell ELISA using mAb BR-1. As control, a flagellin-specific mAb LA21 was employed. (C) C3b deposition on the surface of *B. burgdorferi* B313/pBR cells incubated with 10% NHS was determined using a whole cell ELISA as described above. Values represent the mean of triplicates±SEM. *, P<0.0001. To investigate serum susceptibility to human serum *B. recurrentis* A1 (D), *B.burgdorferi* B313 and transformed *B. burgdorferi* B313/pBR cells (E) were incubated with the indicated concentrations of NHS (dashed line) or heat-inactivated serum (solid line) at 30°C for 3 days. Cells were stained with a nucleic acid dye and the relative growth was determined by measurement of the fluorescence intensities. Values represent the mean±SEM of a single experiment performed in triplicate that is representative of three independent experiments. *, P<0.01; **, P<0.001; ***P<0.0001.

To further verify the significance of HcpA of *B. recurrentis* for complement resistance and removal of C3b from the bacterial surface the serum-sensitive *B. burgdorferi* B313 strain, lacking CFH and CFHL-1 binding proteins was transformed with the shuttle vector pBR containing the complete *hcpA* gene (*B. burgdorferi* B313/pBR). Expression of HcpA was determined by Western blot analysis using an HcpA-specific mAb, BR-1. *B. recurrentis* A1 and the transformed B313/pBR isolate, but not the CFH and CFHL-1-deficient *B. burgdorferi* B313 mutant, expressed HcpA (Fig. 9A, upper panel). HcpA expression on the cell surface of transformed *B. burgdorferi* B313 strains was also detected by whole cell ELISA (Fig. 4B). Moreover, the expressed HcpA protein bound CFH as confirmed by ligand affinity blotting (Fig. 9A, middle panel). In addition, we demonstrate that HcpA expressed on the surface of transformed *B. burgdorferi* B313/pBR cells promotes degradation of deposited C3b (Fig. 9C). To compare the susceptibility of *B. recurrentis* A1, B313 and B313/pBR to complement-mediated killing, the three strains were subjected to a human serum sensitivity assay. Accordingly, *B. burgdorferi* B313/pBR, *B. recurrentis* A1, and *B. burgdorferi* B313 were incubated in NHS or heat-inactivated serum for up to three days. Spirochetal growth was monitored by uptake of a nucleic acid dye. As shown in Figure 9D, *B. recurrentis* A1 readily multiplied during the 72 h time interval in normal human serum, demonstrating the pronounced resistance to human serum of louse-borne relapsing fever spirochetes. Serum-sensitive *B. burgdorferi* B313 as well as B313 spirochetes containing the shuttle vector alone (data not shown) did not grow under similar conditions, suggesting their susceptibility to complement-mediated lysis (Fig. 9E). In contrast, B313/pBR expressing HcpA survived and multiplied in human serum. *B. burgdorferi* B313 and the transformed B313/pBR isolate showed similar growth rates when cultured in heat-inactivated human serum. These findings strongly suggest that HcpA is required for resistance of *B. recurrentis* to complement-mediated killing.

## Discussion

The present results demonstrate for the first time that the etiologic agent of louse-borne relapsing fever, *B. recurrentis*, express a novel multifunctional surface lipoprotein, termed HcpA. By exploiting host proteins, HcpA simultaneously confers resistance to complement attack and opsonization, and in addition, imparts an increased potential to invade host tissues. We show that *B. recurrentis* can bind via its surface expressed HcpA molecule to human complement regulators, i.e. CFH as well as CFHR-1, and in parallel to plasminogen/plasmin. The finding that host-derived factors retain their functional activities, when simultaneously bound to the surfaces of the pathogen underscores the high virulence potential of *B. recurrentis* and makes HcpA a promising target for therapeutic treatment of severe louse-borne relapsing fever [21], [34], [35], [36], [37], [38].

Our data extent previous findings on a related CFH receptor expressed by tick-borne relapsing fever *B. hermsii* spirochetes, including the observation that surface bound CFH facilitates factor I-mediated cleavage of C3b and is critical for immune evasion of these and other pathogens [8], [9]. In fact, CFH binding has also been reported for a number of human pathogens, such as *S. pyogenes* (group A streptococcus) [39], *Neisseria gonorrhoeae* [40], *S. pneumoniae* [41], [42], *Borrelia burgdorferi* [24], [43], *Candida albicans* [44] and other relapsing fever spirochetes [9], [45], [46]. Recombinant HcpA specifically binds CFH and/or CFHR-1 and in addition plasminogen, however via different binding domains. The CFH binding site of HcpA was determined by using mutants with either N-terminal (HcpA_48-175_, HcpA_78-175_) or C-terminal (HcpA_18-145_, HcpA_18-115_) truncations. All mutant of HcpA caused complete abrogation of CFH binding. These data suggest that the determinants required for CFH binding are defined by conformation rather than contiguous linear elements. This finding is reminiscent of the observed CFH binding capabilities for *B. burgdorferi* BbCRASP-1 and BbCRASP-3 [11], [32], [46], [47]. In contrast, binding of PLG to HcpA was not affected by any of the indicated truncations of the HcpA protein, demonstrating that PLG and CFH interact with distinct HcpA domains and suggest that both host proteins can bind simultaneously to HcpA. This assumption is supported by our findings that PLG and CFH bind independently and coordinately to immobilized HcpA and do not compete with each other. Due to the efficient concurrent binding of CFH and PLG to immobilized HcpA it is to be expected that both host proteins similarly bind to HcpA expressed on the outer surface of *B. recurrentis*. Further studies are needed to elucidate the possibility that binding of CFH and PLG to the surface of *B. recurrentis* may be involved in certain pathologies of the central nervous system.

Plasma adsorption experiments and surface plasmon resonance analyses clearly demonstrate that binding of CFH to HcpA is exclusively associated with its C-terminal domain, SCR20. This finding is further substantiated by the fact that HcpA bound CFHR-1, another member of the factor H family that exhibits an almost identical (97%) C-terminal short consensus repeat domain [7]. Thus, the ability of the pathogen to coordinately bind CFH and/or CFHR-1 to HcpA most probably adds to the virulence of *B. recurrentis* by establishing its resistance to complement attack, even under conditions when the human complement regulators are differentially regulated.

Most recently, it was demonstrated that CFHR-1 is an inhibitor of the alternative complement pathway that binds to C3b, inhibits the C5 convertase activity and interferes with C5b surface deposition and membrane attack complex formation (Heinen et al., unpublished). In this context it is of interest that HcpA is also capable of binding another member of the factor H family, i.e. CFHR-2, whose function is yet to be disclosed [7]. The present data show that HcpA-bound CFH retains its regulatory capacity and controls both C3b deposition and C3-convertase activity, resulting in enhanced complement regulatory activity. This process is expected to increase resistance of louse-borne relapsing fever spirochetes to complement attack, an assumption supported by our previous findings with *B. hermsii*.

There is ample evidence suggesting that binding of PLG to bacterial surfaces, including spirochetes, is critical for their invasive potential and persistance [9], [17], [48]. *B. recurrentis* binds PLG and upon processing to enzymatically active plasmin by human uPA the surface bound protease is shown to degrade the physiological substrate fibrinogen. Together with the finding that PLG-coated *B. recurrentis* also bind to the PLG receptors on endothelium cells, our results suggest that spirochetes exploit their increased proteolytic capacity to breach tight junctions of endothelium, cross basement membranes, and to initiate patho-physiological processes in the affected organs [48]. The finding that in accordance to the related Lyme disease spirochetes, *B. recurrentis* bind PLG and can disseminate from the blood to many distant organs, including the brain, supports the assumption that similar mechanisms could be involved [47].

It was recently shown that *Staphylococcus aureus* employs host plasmin to degrade both, surface-bound IgG as well as C3b, and thereby confers resistance to innate and adaptive immune defense processes [49]. Our corresponding finding that HcpA bound plasmin leads to a decrease of C3b molecules on the surface of *B. recurrentis* may represent a novel mechanism, by which spirochetes interfere with C3b deposition on their cell surface and moreover could escape phagocytosis. In line with this assumption, transformed *B. burgdorferi* B313/pBR cells exhibit a significant reduction of C3b molecules deposited on their surface as compared to the parental *B. burgdorferi* B313 strain. Depleted C3b at the bacterial surface correlates with lowering phagocytic activity of human neutrophils [49]. Modulation of opsonic molecules such as C3b seems an ideal strategy for bacterial survival and may account for the extraordinary virulence of *B. recurrentis*. Furthermore, it was recently demonstrated that *B. recurrentis* acquire C4b-binding protein, another regulator of the classical pathway of complement activation on their surface [21]. However, in preliminary experiments binding of HcpA to C4b-binding protein could not be observed. In contrast, a novel 45 kDa protein was shown to strongly interact with C4b-binding protein suggesting that in *B. recurrentis* at least two different receptors for binding of the two complement regulators, CFH and C4b-binding protein, are expressed (unpublished).

Although the biological significance of HcpA expression by *B. recurrentis* has still to be elucidated, the present finding that the *B. burgdorferi* B313 mutant, deficient in CFH receptors, resists complement-mediated killing and following transfection with *hcpA* strongly suggests the involvement of HcpA in immune evasion of *B. recurrentis*. Recently, ectopic expression of the *B. burgdorferi* BbCRASP-1 lipoprotein on the surface of a serum-sensitive Borrelia strain imparts resistance of the transformed isolate to human serum [12], [30], [50]. In the murine model of Lyme disease the precise role of another complement binding protein, BbCRASP-2, was explored and the results of this study suggested that BbCRASP-2 function is dispensable for infectivity [51]. However, studies of the biology of *B. recurrentis* and louse-borne relapsing fever have been hampered by the lack of an animal model. Further investigations are needed to fully examine the complex interplay between *B. recurrentis*, serum sensitivity, and the role of HcpA in the pathogenesis of the disease.

In summary, this is the first study showing the simultaneous and non-competitive binding of CFH and PLG to the outer surface protein HcpA of *B. recurrentis*, the agent of louse-borne relapsing fever. Our finding that HcpA is a multifunctional virulence factor with the potential to simultaneously mediate innate immune evasion and degradation of extracellular matrix components significantly adds to our understanding of the pathological processes underlying louse-borne relapsing fever. Moreover, the presented data support the concept of exploitation of host factors as suitable survival strategies.
